# Age-related differences in the clinical and microbiological profile of pediatric microbial keratitis in Eastern India

**DOI:** 10.1371/journal.pone.0359943

**Published:** 2026-10-14

**Authors:** Rakhi Kusumesh, Nancy Panday, Lalan Kumar Arya, Rizwan Ahmar, Anita Ambasta, Anita Kumari, Namrata Kumari, Bibhuti Prassan Sinha, Nilesh Mohan

**Affiliations:** 1 Cornea and ocular surface services, Regional Institute of Ophthalmology, Indira Gandhi Institute of Medical Sciences, Patna, Bihar, India; 2 Microbiology services, Regional Institute of Ophthalmology, Indira Gandhi Institute of Medical Sciences, Patna, Bihar, India; 3 Department of paediatrics, Indira Gandhi Institute of Medical Sciences, Patna, Bihar, India; 4 Community services, Regional Institute of Ophthalmology, Indira Gandhi Institute of Medical Sciences, Patna, Bihar, India; 5 Department of microbiology, Indira Gandhi Institute of Medical Sciences, Patna, Bihar, India; 6 Glaucoma services, Regional Institute of Ophthalmology, Indira Gandhi Institute of Medical Sciences, Patna, Bihar, India; University of Illinois at Chicago, UNITED STATES OF AMERICA

## Abstract

**Purpose:**

To evaluate the demographics, predisposing factors, microbiological profile, management, and outcomes of pediatric microbial keratitis in Eastern India, with particular emphasis on age-related differences.

**Materials and methods:**

This prospective study included children with clinically diagnosed microbial keratitis between April 2024 and September 2025. Clinical, microbiological, nutritional, treatment, and outcome data were recorded. Children were categorized into two age groups: ≤ 5 years and >5 years for comparative analysis.

**Results:**

Ninety-nine eyes of 86 children were analyzed. The mean age was 5.9 ± 4.8 years, and 42 of 86 children were aged ≤5 years. Bilateral keratitis was observed exclusively in children ≤5 years (p < 0.05). Systemic risk factors such as undernutrition and vitamin A deficiency, diarrhea, and fever were significantly more common in children ≤5 years; in contrast, corneal injury was the predominant risk factor in children >5 years. Mean duration of presentation, ulcer size, and ulcer severity did not differ significantly between the two age groups, although perforation at presentation was more frequent in children >5 years. Fungal keratitis was significantly more common in children >5 years, whereas unidentified cases were more frequent in ≤5 years. Corneal ulcers resolved with medical therapy alone in 59.6% of eyes. Surgery was required in 40.4% of eyes overall, including 18.2% of eyes in children ≤5 years and 22.2% in children >5 years. At 3 months, corneal opacity was the most common outcome.

**Conclusion:**

Pediatric microbial keratitis showed clear age-related differences. Younger children were more often affected by systemic and nutritional vulnerability with predominantly sterile or bacterial keratitis, whereas older children had more trauma-related and fungal disease. Early referral, improved nutritional support, and timely globe-preserving management may reduce visual loss.

**Conference presentation:**

This research work has been selected for presentation at ARVO 2026, Denver, Colo. May 3 - 7

## Introduction

Microbial keratitis (MK) is a vision-threatening ocular condition that may result in corneal scarring and visual disability [1]. Although MK is less common in children than in adults, pediatric cases account for approximately 13%–36.7% of all keratitis cases [2–5]. The impact is often greater in this age group because of the higher risk of amblyopia and poor visual outcome associated with delayed presentation, difficulty in obtaining history and limited cooperation during examination. Therefore, although prompt recognition and treatment are challenging, it is essential to preserve vision and prevent blindness in pediatric patients.

The etiology of pediatric MK is diverse and influenced by multiple factors. Trauma is the leading risk factor in developing countries, whereas contact lens wear predominates in developed regions [6–8]. Variations in predisposing factors, microbial spectrum, and patient demographics significantly affect presentation and outcomes. While several studies worldwide have described the epidemiology, risk factors, and microbiological profiles of pediatric MK, these often differ across the regions and may change over time [9–13]. Moreover, existing reports are constrained by small sample sizes, methodological heterogeneity, and inconsistent management protocols. In addition, age-related differences in risk factors and outcomes within the pediatric population have not been adequately studied and data from Eastern India remain scarce. Therefore, this study aimed to analyze the demographics, risk factors, microbial spectrum, and outcomes of pediatric microbial keratitis in Eastern India with emphasis on age-related differences in disease profile and outcome.

## Materials and methods

### Study design and setting

This prospective study was conducted at a tertiary eye care center in Eastern India over 18 months from April 2024 to September 2025 (S1 Protocol). Approval was obtained from the Institutional Ethics Committee (1524/IEC/IGIMS/24) and the study adhered to the tenets of the Declaration of Helsinki.

### Study population

All consecutive children with clinically diagnosed microbial keratitis, defined by the presence of a corneal stromal infiltrate with an overlying epithelial defect with or without hypopyon, were recruited from the cornea clinic and emergency services. Written informed consent was obtained from parents or guardians. Exclusion criteria included refusal to participate (n = 2), isolated suspected viral keratitis (n = 12), keratitis with endophthalmitis (n = 5), and patients lost to follow-up (n = 13).

### Clinical evaluation

A detailed history and comprehensive ocular examination were performed in all cases. Sociodemographic details, onset and duration of symptoms, predisposing factors, microbiological profile, antibiotic susceptibility, treatment course, and outcome were recorded. Relevant systemic comorbidities and medications along with ocular treatment received before and at presentation were documented. Clinical evaluation included best corrected visual acuity (BCVA) on snellen chart or picture chart in verbal children or fixation and following of light in preverbal children. Slit-lamp biomicroscopy was performed to assess ulcer size, depth, location (central, paracentral, or peripheral), and presence of hypopyon. Intraocular pressure was assessed digitally. Ulcer severity was graded according to modified Jone’s criteria [14]. In eyes with an obscured posterior segment, ultrasonography was performed to exclude endophthalmitis.

### Microbiological workup

Corneal scrapings were obtained under topical anesthesia, sedation, or general anesthesia depending on the child’s cooperation. For each patient, all laboratory procedures were performed under standard protocols. Multiple scrapings were collected for direct microscopy and culture. Smears were prepared for Gram stain and 10% potassium hydroxide wet mount. Sequential scrapings were inoculated onto 10% sheep blood agar, chocolate agar, Sabouraud dextrose agar. Very small ulcers underwent smear examination only, whereas ulcers with a retained foreign body underwent culture alone, with the foreign body plated directly on media. A positive culture was defined as the growth of the same organism on two or more media, confluent growth at the site of inoculation on one solid medium, growth in one medium consistent with direct microscopy findings, or growth of the same organism on repeated corneal scrapings. Antibiotic susceptibility for bacterial isolates was performed using the Kirby–Bauer disc diffusion method in accordance with the guidelines outlined by the Clinical and Laboratory Standards Institute (CLSI) [15]. Susceptibility for fungal isolates could not be performed due to the unavailability of the necessary facility.

### Immunization and nutritional assessment

Immunization status was determined using the Government of India’s National Immunization Schedule [16]. Children were categorized as completely immunized if they had received all age-appropriate vaccines, and incompletely immunized if they had received none or only partial vaccination.

Nutritional status was assessed using the weight-for-height/ weight-for-length based on WHO growth standards [17]. Those above −2 SD from the median were considered to have normal nutrition whereas those below this cutoff were classified as undernutrition**.** For children with systemic associations or suspected undernutrition, a paediatric consultation was sought, and vitamin A supplementation was provided according to WHO guidelines following serum retinol assessment in cases of suspected vitamin A deficiency (VAD) [18]. In children with suspected VAD, Serum retinol levels were measured at admission, prior to treatment initiation, using High-Performance Liquid Chromatography (HPLC). VAD was defined as serum retinol <0.20 mg/L (<0.70 µmol/L) per WHO criteria.

### Treatment protocol

The initial treatment protocol consisted of empirical hourly instillation of broad-spectrum topical antibiotics. Treatment was subsequently modified according to clinical response and microbiological results. When fungi were identified, 5% natamycin drops were added every hour during the day and every 2 hours at night, in addition to antibacterial therapy. Supportive treatment included cycloplegics and topical antiglaucoma agents as needed. Surgical interventions such as cyanoacrylate glue with bandage contact lens (BCL) application, tenon’s patch graft (TPG), or therapeutic penetrating keratoplasty (TPK) were undertaken for cases with full thickness or impending perforation.

### Outcome measures

Patients were monitored daily during the active phase and followed for 3 months. At the end of 3 months, successful outcome was defined as complete resolution of keratitis with corneal opacity, with or without prior surgical intervention, indicating successful control of infection, preservation of globe integrity, and potential for future visual rehabilitation. Poor outcome was defined as anterior staphyloma, phthisis bulbi, or primary graft failure during the study period. Anterior staphyloma and phthisis bulbi represent permanent structural failure with irreversible loss of ocular function. Primary graft failure was included because it indicated failure of visual rehabilitation during the study period, although regrafting may offer future visual potential.

### Statistical analysis

A Microsoft Excel data spreadsheet (Microsoft Corp, Redmond, WA) was used for data entry. The IBM SPSS statistical software program (version 26) was used for all analysis. Frequencies and percentages were used to present categorical variables and continuous variables were presented as mean±SD. Continuous variables were compared using the Wilcoxon–Mann–Whitney U test or 2-tailed independent sample t test. Categorical variables were compared using the Chi-square test or Fisher exact test**.** To identify independent predictors of surgical intervention, perforation at presentation, and poor outcome, multivariable binary logistic regression was performed. The number of variables was limited according to the number of outcome events. Results are reported as odds ratio (OR)/adjusted odds ratio (aOR) with 95% confidence intervals where relevant. P < 0.05 was considered statistically significant.

## Results

### Demographics

A total of 99 eyes from 86 pediatric patients with clinically diagnosed microbial keratitis were analyzed. The mean age was 5.9 ± 4.8 years, with a median of 4.0 years (IQR: 1.0–10.75; range: 8 days–14 years). 50 (58.1%) children were male and 36 (41.9%) female, male-to-female ratio of 1.39:1. Patients were divided into two age groups:  ≤ 5 years and >5 years, based on recognized developmental differences in communication ability, cooperation during examination, and nutritional vulnerability, which may influence disease presentation, management, and outcomes (S2 Dataset Study dataset). Of the 86 children, 42 were aged ≤5 years and 44 were aged >5 years. Among the children aged ≤5 years, 28 were infants. Unilateral keratitis was observed in 73 patients (84.9%) (Fig 1), while 13 (15.1%) had bilateral involvement (Fig 2). Immunization was complete in 58 (67.4%) and incomplete in 28 (32.6%). Most patients belonged to the lower middle class (39; 45.3%) and upper lower class (28; 32.6%), followed by upper middle 17 (19.8%) and lower 2 (2.3%); none were from the upper class based on revised Kuppuswamy socioeconomic status class classification [19]. The majority resided in rural areas (65.1%). Nutritional evaluation showed that 62 children (72.1%) were within normal limits, while 24 (27.9%) were undernourished. More than half of the infants (67.8%) were bottle-fed with diluted cow’s milk. The demographic, socioeconomic, nutritional, and immunization profile of the study population by age groups is summarized in Table 1.

**Fig 1:**
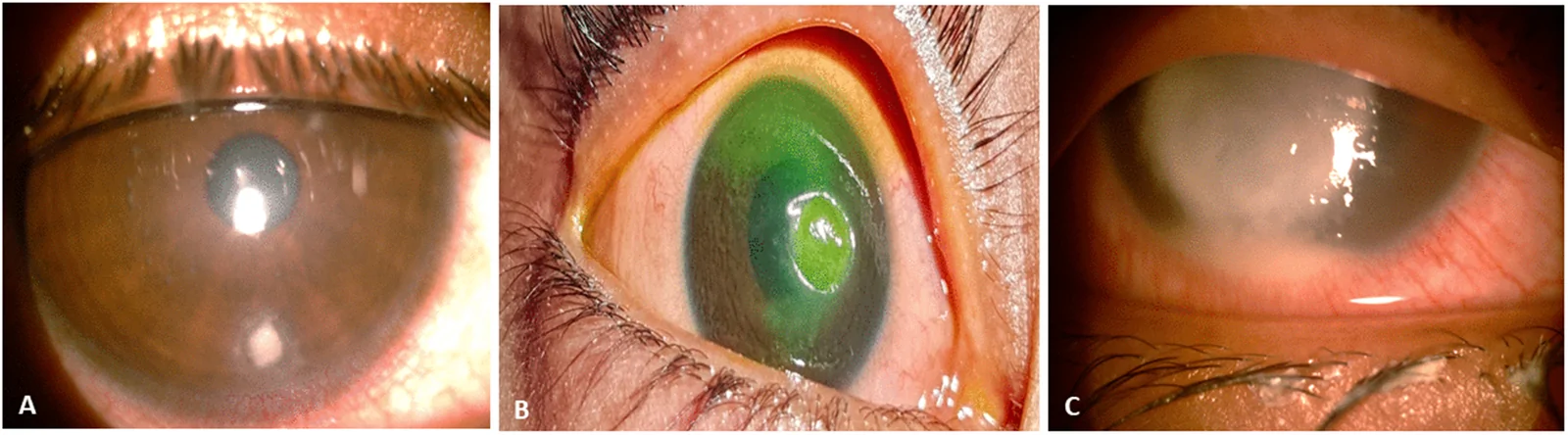
Clinical photographs showing pediatric microbial keratitis graded according to modified Jones criteria: (A) mild keratitis with superficial paracentral corneal involvement; (B) moderate keratitis with a central ulcer extending up to 50% of the corneal thickness; and (C) severe keratitis with stromal involvement exceeding 50% and associated hypopyon.

**Fig 2:**
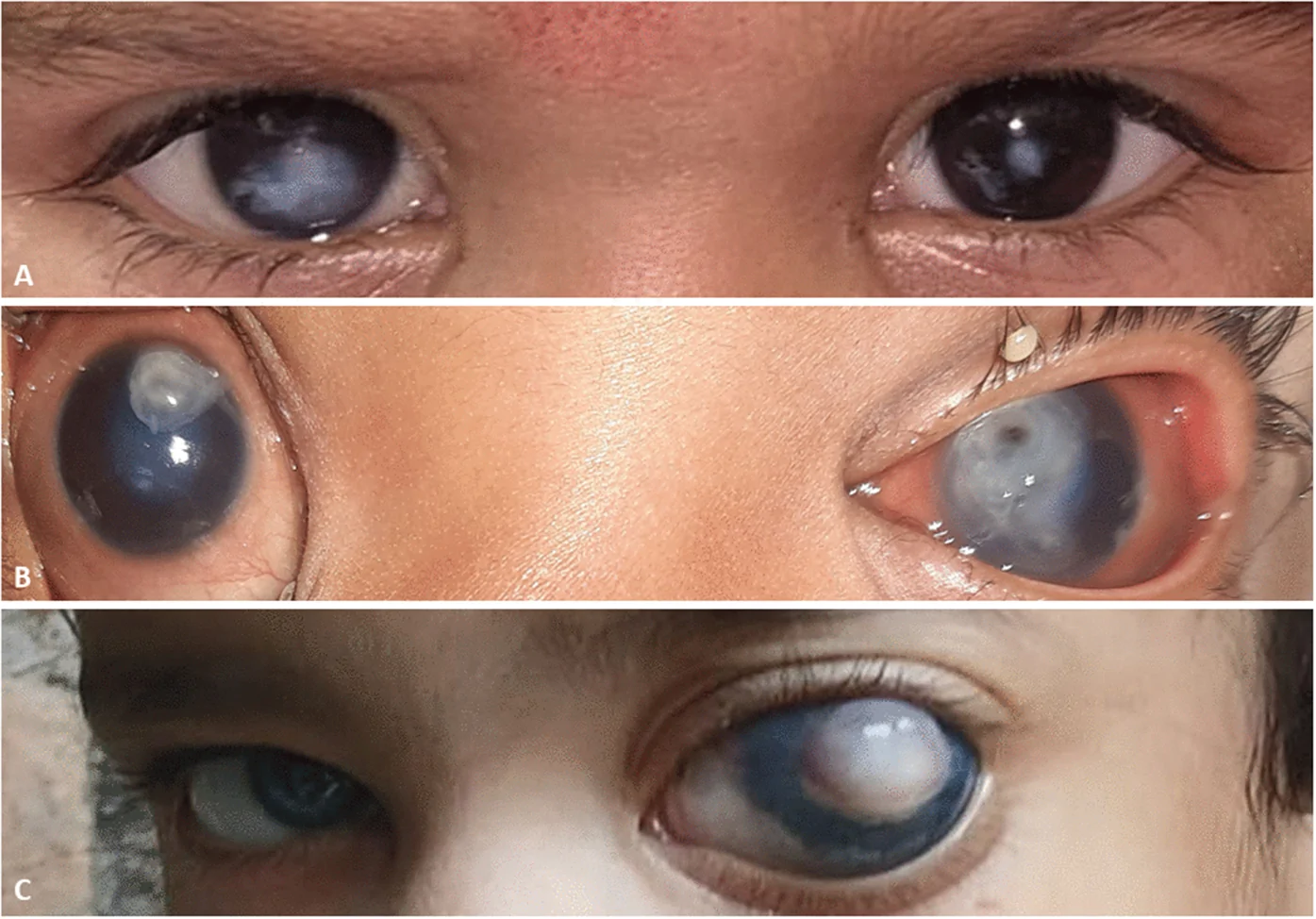
Clinical photographs of bilateral keratitis associated with vitamin A deficiency: (A) bilateral healed keratitis with corneal opacity; (B) bilateral perforated corneal ulcers in a 7-month-old boy; and (C) the right eye at 5 months after therapeutic penetrating keratoplasty and the left eye with anterior staphyloma and secondary glaucoma.

**Table 1:** Demographic, socioeconomic, nutritional, and immunization profile of pediatric patients (n = 86) by age group.

Variables	Total children (n=86)	≤5 Years children (n=42)	>5 Years children (n=44)	P value
Number of patients (number of eyes)	86 (99 eyes)	42 (55 eyes)	44 (44 eyes)	
Mean age ±std	5.94 ± 4.78 years	1.50 ± 1.62 years	11.52 ± 2.45 years	<0.05
Median (IQR)	4.0 years (1.0–10.75)	0.75 years (0.25–2.50)	12 years (8.0–12.0)	
Range of age	8 days to 14 years	0.02–5.0 years	6.0–14.0 years	
Gender				0.855
Male	50 (58.1%)	24 (57.1%)	26 (59.1%)	
Female	36 (41.7%)	18 (42.86%)	18 (40.9%)	
Laterality				<0.05
Unilateral	73 (84.9%)	29 (69.1%)	44 (100%)	
Bilateral	13 (15.1%)	13 (30.9%)	00	
Socio-economic status				0.025
Upper middle	17 (19.8%)	11 (26.2%)	06 (13.64%)	
Lower middle	39 (45.3%)	21 (50.0%)	18 (40.91%)	
Upper lower	28 (32.6%)	08 (19.1%)	20 (45.45%)	
Lower	02 (2.3%)	02 (4.8%)	00 (0.00%)	
Nutrition status				<0.05
Normal	62 (72.1%)	24 (57.1%)	38 (86.36%)	
Undernutrition	24 (27.9%)	18 (42.9%)	06 (13.64%)	
Immunization				0.045
Complete	58 (67.4%)	24 (57.1%)	34 (77.27%)	
Incomplete	28 (32.6%)	18 (42.9%)	10 (22.73%)	

### Predisposing factors

The age-wise distribution of predisposing factors is summarized in Table 2. Among predisposing factors, systemic illness emerged as the most common factor in children ≤5 years in 57.1% of cases, compared with only 9.1% in children >5 years (p < 0.05). The most common systemic associations were diarrhoea and VAD, with the majority of children ≤5 years exhibited more than one systemic risk factor. VAD was present in 42.9% of children ≤5 years at presentation, including 73% of infants (OR = 18.83; 95% CI: 4.14–85.57). At presentation, the median serum retinol level was 0.03 mg/L (IQR 0.02–0.13), which increased to 0.22 mg/L (IQR 0.20–0.27) following supplementation. Undernutrition was more common in children ≤5 years (42.9%), who had nearly five times higher odds of being undernourished compared with children >5 years (OR = 4.75; 95% CI: 1.65–13.65). In contrast, among children >5 years, corneal injury was the predominant risk factor (81.8%).

**Table 2:** Predisposing factors associated with keratitis in 86 patients across the age groups.

Variables	≤5 Years children (n=42)	>5 Years children (n=44)	P value
**Ocular factors**	10 (23.8%) *	37 (84.1%) *	<0.05
Corneal Injury	02 (4.8%)	26 (59.1%)	<0.05
Dust	02 (4.8%)	03 (6.8%)	<0.05
Foreign Body	01 (2.4%)	03 (6.8%)	0.746
Bee Sting	01 (2.4%)	01 (1.2%)	1.00
Allergic Conjunctivitis	00	01 (1.2%)	0.273
Corneal Anaesthesia	01 (2.4%)	01 (1.2%)	0.273
Kajal	01 (2.4%)	00	0.273
NLDO	02 (4.8%)	01 (1.2%)	1.000
Ocular Rosacea	00	01 (1.2%)	
**Systemic factors**	24 (57.1%) *	4 (9.1%) *	<0.05
Vitamin A deficiency	18 (42.9%)	2 (4.5%)	<0.05
Diarrhoea	19 (45.2%)	00	<0.05
Malnutrition	09 (21.4%)	2 (4.5%)	0.019
Intrahepatic cholestasis	02 (4.8%)	0 (0.0%)	0.236
Fever	16 (38.1%)	0 (0.0%)	0.488
Type 1DM	00	1 (2.3%)	1.000
SJS Sequelae	00	1 (2.3%)	1.000
**None or unidentified**	08 (19.1%)	03 (6.8%)	0.113

* Percentages were calculated using the total number of children in each age group as the denominator. As some children had more than one predisposing factor, the percentages for individual risk factors may not total 100%

Further analysis showed that bilateral involvement was significantly more frequent among undernourished than normally nourished children (37.5% vs 6.5%; P = 0.001) and among incompletely than completely immunized children (28.6% vs 8.6%; P = 0.024). Incomplete immunization was also more common among undernourished children (50.0% vs 25.8%; P = 0.042). Overall, 12 children (14.0%) were both undernourished and incompletely immunized, of whom 5 (41.7%) had bilateral keratitis. Bilateral involvement was more frequent in lower socioeconomic groups, although this association did not reach statistical significance (P = 0.053).

### Clinical features and microbial profile

Bilateral keratitis was observed exclusively in children ≤5 years (Fig 2), whereas all children older than 5 years presented with unilateral disease (p < 0.05). Although mean duration of presentation (p = 0.651), ulcer size (p = 0.810), and severity (p = 0.412) did not differ between the two age groups (Fig 1), perforation at presentation was more frequent in children >5 years than in those ≤5 years (31.8% vs 18.2%); however, this difference was not statistically significant (p = 0.116). All eyes in both groups had received prior topical antibiotic, and prior use of antibiotic–corticosteroid combination was significantly more common in children >5 years (p = 0.031).

All 99 eyes underwent microbiological evaluation by smear and culture. Causative organisms were identified in 51.5%, constituting the microbiologically confirmed cases, while 48.5% were negative on both smear and culture (unidentified/sterile). All microbiologically confirmed eyes were smear-positive, of which 36.4% of all eyes were also culture-positive. Microbiological confirmation was higher in children older than 5 years (68.2%) than in those ≤5 years (38.2%; P = 0.004). Fungal keratitis was significantly more common in children >5 years (p < 0.05). Clinical profile, microbiology, management, and outcome by age group are summarized in Tables 3 and 4.

**Table 3:** Clinical characteristics at presentation, management, and outcome by age group.

Variables	≤5 Years (55 eyes)	>5 Years (44 eyes)	P value
Number of patients (number of eyes)	42 (55 eyes)	44 (44 eyes)	
Duration of presentation (Days)	12.15 ± 5.86	12.82 ± 7.00	0.651
Size of ulcer (mm)	5.49 ± 2.22	5.62 ± 2.05	0.810
Grade of ulcer			0.412
Mild	13 (23.6%)	08 (18.2%)	
Moderate	21 (38.8%)	21 (47.7%)	
Severe	21 (38.8%)	15 (34.1%)	
Perforation (at presentation)	10 (18.2%)	14 (31.8%)	0.116
Causative microbe (smear/culture)			
Bacterial	14 (25.5%)	07 (15.9%)	0.341
Fungal	07 (12.7%)	23 (52.3%)	<.05
Unidentified/sterile	34 (61.8%)	14 (31.8%)	0.022
Previous medical history			
Antibiotics	55 (100%)	44 (100%)	
Antibiotics + antifungal	18 (32.7%)	15 (34.1%)	0.705
Antibiotics + corticosteroid	23 (41.8%)	32 (70.5%)	0.031
Medical management only	37 (67.2%)	22 (50%)	0.495
Surgical intervention			0.876
Glue application	9 (16.3%)	8 (18.8%)	
Tenon’s patch graft	2 (3.6%)	1 (2.3%)	
Therapeutic keratoplasty	7 (12.7%)	13 (29.5%)	
Outcome (at 3 months)			0.819
Corneal opacity	42 (76.4%)	30 (68.2%)	
Clear graft	03 (5.4%)	8 (18.8%)	
Failed graft	04 (7.2%)	5 (11.4%)	
Phthisis bulbi	04 (7.3%)	02 (4.5%)	
Secondary glaucoma	11 (20.0%)	08 (18.2%)	
Anterior staphyloma	03 (5.5%)	00	
Pre-Intervention BCVA (Log MAR)		1.49 ± 0.69	
Post-Intervention BCVA (Log MAR)		0.75 ± 0.51	

**Table 4:** Microbiological profile of pediatric microbial keratitis by age group.

Isolated organisms	Total no. of eyes (n=99)	≤5 Years (n=55)	>5 Years (n=44)	P value
**Smear positive**	51 (51.5%)	21 (38.2%)	30 (68.2%)	0.004
**Culture positive**	36 (36.4%)	14 (25.5%)	22 (50%)	0.020
**Unidentified/sterile**	48 (48.48%)	34 (61.8%)	14 (31.8%)	0.022
**Bacterial isolates**	14 (14.1%)	10 (18.1%)	04 (9.1%)	0.252
*Staphylococcus aureus*	04 (4.0%)	03 (5.5%)	1 (2.3%)	
Coagulase-Negative	07 (7.1%)	05 (9.1%)	2 (4.5%)	
*Staphylococci spp*				
*Streptococcus pneumoniae*	01 (1.0%)	01 (1.8%)	0	
*Pseudomonas aeruginosa*	01 (1.0%)	0	1 (2.3%)	
*Serratia spp.*	0	0	0	
*Enterococcus spp.*	01 (1.0%)	01 (1.8%)	0	
*Acinetobacter baumannii*	01 (1.0%)	00	1 (2.3%)	
**Fungal isolates**	22(22.2%)	04 (7.2%)	18 (40.9%)	<0.05
*Aspergillus spp*	16 (16.1%)	03 (5.5%)	13 (29.5%)	
*Fusarium spp*	05 (5.1%)	01 (1.8%)	04 (9.1%)	
*Curvularia spp*	01 (1.0%)	0	01 (2.3%)	

### Management and outcome

Broad-spectrum fluoroquinolones were the mainstay of treatment in bacterial keratitis, whereas topical natamycin was the preferred treatment in fungal keratitis. Gram-positive organisms showed good sensitivity to ciprofloxacin, levofloxacin, vancomycin, tobramycin, gentamicin, and cefoxitin, while gram-negative organisms were generally susceptible to fluoroquinolones, aminoglycosides, and colistin. Corneal ulcers resolved with medical therapy alone in 59.6% of eyes (Fig 2A). Although the number of cases managed medically or surgically did not differ significantly between the two age groups, surgical intervention was required in 32.7% of cases in children ≤5 years and 50% of cases in children >5 years. Among the 99 eyes, 40 (40.4%) required surgical intervention for restoration of globe integrity (Fig 3). Of these, 20 (20.2%) eyes were managed with minor tectonic procedures such as cyanoacrylate glue with BCL application (Fig 3B) and TPG (Fig 3E), whereas the remaining 20 (20.2%) eyes underwent TPK (Fig 2C, Fig 3 D) (Table 3).

**Fig 3:**
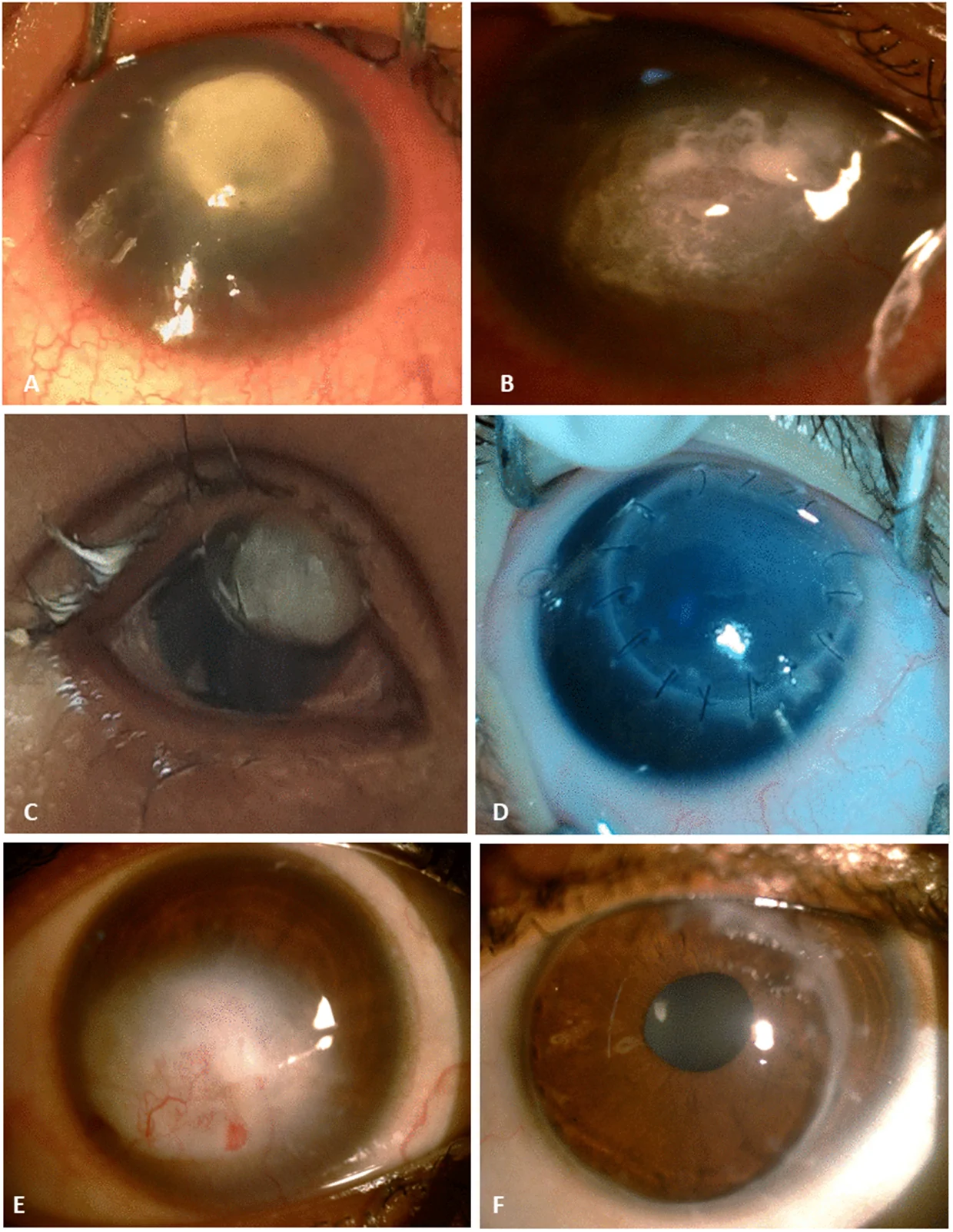
Clinical photographs showing management and outcomes of pediatric microbial keratitis: (A) non-healing corneal ulcer with impending perforation; (B) same eye after cyanoacrylate glue application with bandage contact lens in a 6-year-old child; (C) perforated corneal ulcer in a 2-year-old child; (D) same eye at 1 month after therapeutic penetrating keratoplasty; (E) well-incorporated Tenon’s patch graft in a perforated corneal ulcer; and (F) eye at 3 months after therapeutic penetrating keratoplasty in a 6-month-old child.

On multivariable analysis, larger ulcer size and longer duration of keratitis were independently associated with surgical intervention. Each 1-mm increase in ulcer size increased the odds of surgical intervention by 73% (aOR 1.73; 95% CI 1.05–2.85; p = 0.032), while each additional day of disease increased the odds by 12% (aOR 1.12; 95% CI 1.03–1.22; p = 0.009). Severe ulcer grade and longer disease duration were independently associated with perforation at presentation. Severe ulcers had more than sixfold higher odds of perforation (aOR 6.32; 95% CI 2.14–18.63; p = 0.001), and each additional day of disease increased the odds by 14% (aOR 1.14; 95% CI 1.05–1.24; p = 0.002). Severe ulcer grade (aOR 7.43; 95% CI 2.08–26.49; p = 0.002) and incomplete immunization (aOR 4.25; 95% CI 1.28–14.07; p = 0.018) were independently associated with poor outcome at 3 months.

At 3 months, corneal opacity was the most common outcome in both age groups (Fig 2A). Following TPK, clear graft was seen more often in children >5 years (18.8%) (Fig 3F) than in those ≤5 years (5.4%). Anterior staphyloma was observed only in children ≤5 years (Fig 2C). Phthisis bulbi and secondary glaucoma were seen in both groups, with no significant difference in the overall outcome profile (p = 0.819). Visual acuity assessment was possible only in children >5 years, and mean BCVA improved from 1.49 ± 0.69 LogMAR at presentation to 0.75 ± 0.51 LogMAR at 3 months of follow-up.

## Discussion

This prospective study aimed to evaluate the demographics, predisposing factors, microbiological profile, and outcomes of pediatric microbial keratitis in Eastern India, with a specific focus on age-related differences between children ≤5 years and those older than 5 years. Unlike our previous retrospective study, which evaluated microbial keratitis across all age groups and focused mainly on regional epidemiology and microbiology, the present study exclusively included children and systematically assessed pediatric-specific factors such as nutritional status, vitamin A deficiency, immunization, bilateral involvement, management, and outcomes. To our knowledge, no previous study has specifically evaluated age-related differences in microbial keratitis within the pediatric population. The study enrolled 86 children (99 eyes) over 18 months and yielded several clinically important findings.

The age distribution in the present study showed a distinctly younger pediatric profile, with a mean age of 5.9 ± 4.8 years and nearly half of the cases occurring in children ≤5 years, in contrast to other studies where the mean age ranged from 9 to 13 years. Chandratreya et al. reported a mean age of 10.81 years from South India, with 50.7% of patients aged between 11 and 16 years [20]. In contrast, Vajpayee et al. reported findings closer to the present study, observing a younger pediatric population and an association with lower socioeconomic status, poor nutrition, and incomplete immunization [21]. Most children in our cohort belonged to rural areas and lower socioeconomic groups, highlighting the role of social and environmental factors in disease occurrence. In addition, a male predominance (58.1%) was observed, consistent with previous studies, and may be related to greater outdoor exposure and a higher risk of ocular injury, especially in older children [10,20–23].

This study demonstrated an age-related difference in both clinical presentation and predisposing factors. Children ≤5 years were significantly more likely to present with bilateral keratitis, whereas all children >5 years had unilateral disease (p < 0.05). Children ≤5 years had nearly fivefold higher odds of being undernourished, and systemic risk factors such as diarrhea, fever, malnutrition, and VAD were markedly more frequent in this group (OR 13.33, 95% CI 4.40–50.58; p < 0.001). Similar observations have been reported by Kunimoto et al., Jhanji et al., and Vajpayee et al., who highlighted the important contribution of systemic and nutritional vulnerability in younger children with keratitis [21,24,25].

VAD emerged as an important finding in this study. Despite supplementation programs, it remains an important cause of childhood blindness in children <5 years, with reported prevalence up to 30% in vulnerable population [26–29]. In our study, 43% of children <5 years had VAD, with a median serum retinol level of 0.03 mg/L (IQR 0.02–0.13) at presentation. The high burden of VAD in these infants may be partly related to nutritional vulnerability caused by inappropriate feeding practices including bottle-feeding with diluted cow’s milk. The association of VAD with diarrhea and febrile illness is important as these illnesses can further deplete vitamin A stores and trigger acute ocular disease [30]. This predominance of VAD in younger children is also reported by the recent study by Baker et al., in which the median age was 2.78 years, 95% of children had bilateral involvement, and more than half showed keratomalacia with or without bacterial keratitis [31].

Vitamin A is essential for maintaining corneal and conjunctival epithelial integrity and normal mucus-secreting function. Its deficiency causes ocular-surface dryness and xerosis with loss of local corneal defense. Severe deficiency may lead to ulceration and keratomalacia, which can become secondarily infected and result in stromal melting, perforation, and scarring [32–35]. Baker et al., in a retrospective study of children with VAD-related corneal disease requiring keratoplasty, demonstrated the severe consequences of this process including keratomalacia, corneal perforation, anterior staphyloma, phthisis, and extensive corneal scarring [31].

Undernutrition was also more frequent in younger children and is closely linked to VAD. Together, undernutrition and VAD may impair systemic immunity, epithelial integrity, and tissue healing, thereby increasing susceptibility to microbial keratitis and its severe complications. In addition, other micronutrients such as zinc, iron, folate, vitamin B12, and vitamin C also contribute to normal immune function and host resistance to infection [36]. Incomplete immunization, observed in 32.6% of children in our study, may further reflect this systemic vulnerability. Among vaccine-preventable illnesses, measles is particularly important, as it can worsen VAD, suppress local and systemic immunity, and predispose to corneal ulceration and secondary microbial infection [30,37].

In contrast, children >5 years predominantly had ocular risk factors, especially corneal injury consistent with other studies from developing countries where trauma was a major predisposing factor in older children [10,20–23]. This age-related difference in trauma exposure may also explain the predominance of fungal over bacterial keratitis in older children. Injury with vegetative matter, an important risk factor for fungal keratitis in tropical regions, is more likely in school-going children with greater outdoor exposure. In our cases, contact lens–related keratitis was not observed, unlike studies from developed countries where the incidence of trauma is decreasing and contact lens–related keratitis is increasing [38]. Overall, these findings indicate pediatric microbial keratitis is associated with identifiable risk factors, although their pattern varies with age, geography and healthcare facility.

Although ulcer size and severity were not different between two age groups, perforation at presentation was more frequent in children >5 years (31.8% vs 18.2%). This may be explained by the fact that ulcers in older children were more often trauma-related, fungal in etiology, and exposed to prior topical corticosteroids. We also observed that eyes with prior topical corticosteroid use had a greater tendency to develop perforation and poorer outcomes. Similar findings have been reported in our earlier study, in which prior corticosteroid use was associated with perforation requiring immediate surgical intervention in 30.5% of cases [39]. Therefore, non-prescription sale of topical corticosteroids should be more strictly controlled. In this study, as most children were referred after receiving variable treatment elsewhere, details regarding the specific drugs, doses, duration of therapy, and treatment adherence could not be reliably ascertained from the referral records. Delayed presentation was another important finding in both age groups. Children presented after a mean symptom duration of nearly 12 days. This was much longer than the 4.19 days reported in South India and 5 days from North India, in which 78.1% and 64.4% of children, respectively, presented within the first week [13,20]. Chaurasia et al. also observed that keratitis in neonates and very young children may be clinically subtle and therefore easily missed, predisposing to delayed diagnosis. However, in our setting delay likely reflects limited access to specialist care, delayed referral, and prolonged empirical treatment [40]. Together, these factors may have contributed to relatively advanced ulcer profile observed at presentation in both groups. This highlights the importance of training local healthcare workers to identify ocular emergencies early and ensure timely referral to adequately equipped eye care centers.

The majority of children ≤5 years (62%) had no identifiable organism, whereas children >5 years showed higher microbiological positivity (68%) and more fungal isolates (43%). This indicates age-related differences in disease pattern, with lower microbiological yield in younger children (≤5 years) and a greater fungal burden in older children (>5 years). The predominance of *Aspergillus* followed by *Fusarium* in our cases is consistent with reports from India and other tropical regions [21]. However, studies from South India and rural China have reported *Fusarium* as a common fungal isolate [20,23]. By contrast, neonatal and other pediatric series have reported largely bacterial keratitis with *Pseudomonas aeruginosa* as a common isolate [3,41,42]. In our study, however, bacterial isolates were mainly gram-positive cocci, particularly coagulase-negative staphylococci. Overall, this study demonstrated that smear positivity (51.5%) was higher than culture positivity (36.4%) likely because many children had received prior antimicrobial treatment before referral. A recent publication also observed poor culture growth in referred patients, possibly due to the bacteriostatic effects of topical antibiotics received prior to referral [43].

Empirical therapy is commonly initiated in pediatric microbial keratitis because microbiological workup is often challenging in children. Because our institute is a tertiary referral center, nearly all children had already received topical antibiotics, antifungals, corticosteroids, or their combinations before presentation. Although medical management alone was more common in children ≤5 years and surgery was more frequent in children >5 years, the overall difference was not statistically significant. Despite delayed referral and advanced disease with corneal thinning and frank perforation at presentation, TPK was required in only 20% of cases, compared with reported surgical intervention rates ranging from 0% to 77.6% in previous pediatric series [3,20,21,23,24]. In our series, TPK could often be avoided by using alternative tectonic procedures such as cyanoacrylate glue application and TPG, particularly in sterile ulcers or bacterial ulcers requiring urgent tectonic support. These procedures are especially useful in children because they can be performed under short general anesthesia and help preserve globe integrity while controlling infection and providing time for a definitive vision-restorative procedure. In addition, children tend to have more intense inflammation and earlier corneal vascularization than adults, which favours an early minor tectonic approach in selected cases, especially in settings where donor corneal tissue is scarce.

At 3 months of follow-up, corneal opacity was the most common final outcome, similar to other pediatric series. More than half of the eyes healed with medical therapy alone or with non-keratoplasty tectonic procedures. Among eyes undergoing keratoplasty in our series, nearly half of the grafts became opaque because of high-risk factors such as young age, active inflammation, larger grafts, vascularization, and rejection [44]. Cowden et al. identified corneal perforation, active inflammation or infection, ocular comorbidity, and repeated interventions as predictors of poor prognosis [45]. Although the overall outcome was comparable between age groups, anterior staphyloma was significantly more frequent in infants <1 year, particularly in those with VAD. Overall, our findings indicate that outcome in pediatric microbial keratitis is determined not only by age, but also by nutritional status, severity at presentation, inflammation, and timely intervention.

This study has some limitations. Since it was conducted at a tertiary referral center, many patients had severe and recalcitrant keratitis, the findings may therefore not represent the entire spectrum of pediatric microbial keratitis. In addition, quantitative visual acuity assessment was not possible in many younger children, in whom visual function was assessed qualitatively by fixation and following of light. Therefore, visual outcomes could not be directly compared between age groups. Deprivation amblyopia, particularly in children aged ≤5 years, could not be adequately assessed during the short follow-up period. The 3-month follow-up period also limited assessment of longer-term outcomes including corneal graft survival, progression of corneal scarring, and visual rehabilitation. Another limitation of this study was the lack of advanced microbiological examination techniques for the detection of organisms that are difficult to identify by conventional methods such as in vivo confocal microscopy, polymerase chain reaction, and next‑generation sequencing. However, this study also has important strengths. It is a prospective study with large number of children, with more than half of them being under 5 years of age, and it included both culture-positive and culture-negative cases.

## Conclusion

Pediatric microbial keratitis shows age-related differences in predisposing factors, clinical features, microbiological profile, and outcome. Systemic and nutritional vulnerability, particularly VAD and undernutrition, predominated in younger children (≤5 years) and is associated with a predominance of sterile or bacterial keratitis, whereas trauma-related and fungal keratitis are more common in older children (>5 years). Delayed presentation and prior empirical treatment appear to worsen disease severity at presentation. However, favorable structural outcomes can still be achieved in many eyes with timely medical treatment and selective globe-preserving tectonic procedures. Early referral and improved nutritional support may help reduce visual loss in this population.

## Supporting information

S1 Protocol Approved study proposal for pediatric microbial keratitis research. (DOCX)

S2 Dataset De-identified study dataset. (XLSX)
