# Supplementary material for: Age-related differences in the clinical and microbiological profile of pediatric microbial keratitis in Eastern India
Source: PLoS One. 2026 Oct 14;21(10):e0359943. doi: 10.1371/journal.pone.0359943 (PMC13667804; doi:10.1371/journal.pone.0359943)
Supplement: Supplementary file 1 [file pone.0359943.s003.pdf]

Reviewer's Comments

Manuscript No.: PONE-D-26-21297
Title: Age-related differences in the clinical and microbiological profile of pediatric microbial keratitis in Eastern India
Authors: Rakhi Kusumesh *et al*.

Comments:
The manuscript entitled 'Age-related differences in the clinical and microbiological profile of pediatric microbial keratitis in Eastern India', aims to evaluate the demographics, predisposing factors, microbiological profile, management and outcomes of pediatric microbial keratitis in Eastern India with emphasis on age-related differences.

Following comments need to be addressed at appropriate places in the manuscript:

1. How is this study different from the author's own previous study (Kusumesh et al., IJO, 2023, PMID: 37870015)? Please highlight in terms of novelty, new information, and distinctions.
2. Why were the children categorized in >5 and <5 years? The rationale of choosing 5 years of age as a bar is not clear.
3. Line 41/Materials and methods: 'This prospective study.......**suspected** microbial keratitis.......'. Why were the suspected and not only the confirmed cases considered? Was this suspicion based on clinical or microbiological profiling of patients? Ideally, only the microbiologically and clinically confirmed cases should only be included in this study.
4. Line 48/ Results: How do undernutrition and vitamin A deficiency aid to microbial infections?
5. Line 60: What could be the possible reasons for children with age more than 5 years to get fungal infections than bacterial keratitis?
6. Lines 122-125: Immunization and nutritional assessment: Considering that immunization and nutritional assessment are the important criteria, can the authors name a few vaccines and few nutritional elements which can be critical for occurrence, severity, and/or recurrence of microbial keratitis?
7. Lines 152-154: Results/Demographics: 'A total of 99 eyes from 86 ....patients with **suspected** microbial keratitis'. How many of these were the confirmed (microbiologically) cases?
8. Table 1: What was the interrelationship between gender, laterality, socio-economic status, nutritional status, and immunization status? For example: How many of the confirmed male patients (children) were unilateral, belonging to lower middle socio-economic status, were undernourished and incompletely immunized? Such correlations among different groups may further add to data interpretation and conclusion.
9. Table 3: 'Causative microbe' should preferably come before 'Previous medical history'. Further, were the duration of prescribed antimicrobial drugs and corticosteroid also considered? If yes, what were the dose start and endpoints with respect to date of presentation, onset, and resolution of infection. How many of the patients followed the prescribed treatment regimen?
10. Table 4: Names of bacterial and fungal isolates should follow the standard nomenclature pattern (in italics), as applicable, throughout the manuscript.
