# Supplementary material for: Age-related differences in the clinical and microbiological profile of pediatric microbial keratitis in Eastern India
Source: PLoS One. 2026 Oct 14;21(10):e0359943. doi: 10.1371/journal.pone.0359943 (PMC13667804; doi:10.1371/journal.pone.0359943)
Supplement: Supplementary file 2 [file pone.0359943.s001.docx]

## Research Proposal Multi-Disciplinary Research Unit (MRU)

Indira Gandhi Institute of Medical Sciences, Patna, Bihar

## Project title:

Epidemiological and microbiological profiles of pediatric microbial keratitis in a tertiary eye center in Eastern India (Bihar)

## Specific area of the proposed research:

Ocular infection / Pediatric corneal diseases

## Objectives of the study:

**Primary:**
1. To evaluate the demographic and clinical profile of pediatric microbial keratitis in Eastern India.
2. To identify ocular, systemic, nutritional, and socioeconomic risk factors associated with pediatric microbial keratitis.
3. To analyze the microbiological spectrum of pediatric microbial keratitis.

**Secondary:**
1. To compare clinical presentation, microbiological profile, management strategies, and outcomes between children aged ≤5 years and >5 years.
2. To evaluate anatomical and visual outcomes following medical and surgical treatment.

## Rationale of study:

## Pediatric microbial keratitis is an important cause of preventable corneal blindness in developing countries. Data from Eastern India regarding its risk factors, microbiological profile, management, and outcomes are limited. This study aims to characterize these features and also evaluate age-related differences within the pediatric population.

## Methodology

This will be a prospective observational study to be conducted at the Regional Institute of Ophthalmology, IGIMS, Patna, over a period of 18 months, from April 2024 to September 2025. For comparative analysis, children will be categorized into two age groups: ≤5 years and >5 years.

**Inclusion and exclusion criteria:**
Children aged ≤14 years presenting with clinically diagnosed microbial keratitis, defined as a corneal stromal infiltrate with an overlying epithelial defect with or without hypopyon, will be enrolled after obtaining written informed consent from parents or guardians. Patients with isolated viral keratitis, refusal of consent, loss to follow-up, or endophthalmitis/panophthalmitis will be excluded.

**History:** A detailed history will be recorded in all eligible patients, including duration of symptoms, predisposing factors, prior treatment received, use of topical corticosteroids, access to the nearest health centre, contact lens use, and any history of previous ocular surgery. History of trauma, foreign body exposure, fever, diarrhoea, and any systemic illness will also be documented.

In infants, additional details such as birth weight, gestational age, mode of delivery, neonatal intensive care unit stay or prolonged hospitalization, breastfeeding status, bottle hygiene in formula-fed infants, vaccination status, and nutritional status along with assessment for vitamin A deficiency will be noted.

Maternal antenatal history, including birth order, abortions, fever or rash during pregnancy, and other comorbidities, will also be recorded.

**Examination:** Clinical examination will include slit-lamp evaluation of the affected and fellow eyes for ulcer size, location, depth, presence of perforation, height of hypopyon, and intraocular pressure in cooperative children. Best-corrected visual acuity will be assessed wherever possible. Younger and uncooperative children will be examined under sedation using a portable slit lamp. Visual function in preverbal children will be assessed by fixation and following of light.

**Microbiological examination:** For microbiological evaluation, multiple corneal scrapings will be collected under standard aseptic protocols for direct microscopy and culture. Smears will be prepared for Gram staining and 10% potassium hydroxide wet mount. Corneal scrapings will then be inoculated onto 10% sheep blood agar, Sabouraud’s dextrose agar, non-nutrient agar, and chocolate agar in C-shaped streaks. A culture will be considered positive if the same organism grows on two or more media, shows confluent growth at the site of inoculation on a solid medium, correlates with direct microscopy findings, or is isolated repeatedly from subsequent corneal scrapings.

Antibiotic susceptibility testing for bacterial isolates will be performed by the Kirby–Bauer disc diffusion method in accordance with Clinical and Laboratory Standards Institute guidelines. For the purpose of analysis, isolates showing intermediate or resistant response will be classified as resistant. Fungal susceptibility testing will not be performed because the necessary facility is currently unavailable.

**Expected sample size and feasibility:**

Based on cornea clinic attendance of approximately 150–160 patients per month, with pediatric keratitis constituting nearly 5% of cases, about 7–8 eligible children are expected to be recruited per month. This supports the feasibility of achieving the proposed sample size within the study period.

**Duration of study:** The study duration will be 18 months, including 15 months for patient recruitment and 3 months for data analysis and preparation of the final report.

**Follow-up**

All enrolled children will be followed until complete resolution of keratitis and subsequently for at least 3 months to document anatomical and visual outcomes.

**Outcome measures**

Primary outcomes will include microbiological profile, anatomical outcome, and visual outcome wherever assessable. Anatomical outcome will include healed scar, perforation, graft status, anterior staphyloma, phthisis bulbi, and other structural complications.

**Age-group comparison**

Children will be stratified into two age groups, ≤5 years and >5 years, to assess age-related differences in predisposing factors, microbiological spectrum, clinical presentation, management, and outcomes.

**Data analysis**
Data will be entered into Microsoft Excel and analyzed using SPSS version 26. Continuous variables will be expressed as mean ± standard deviation or median with interquartile range, and categorical variables as frequency and percentage. Appropriate statistical tests will be applied for group comparisons, and a p value <0.05 will be considered statistically significant.

**Institutional support**
The study is feasible because the institute receives a regular load of pediatric keratitis cases through the cornea clinic and has access to standard microbiological facilities required for smear examination, culture, and antibiotic susceptibility testing.
