# Supplementary material for: Age-related differences in the clinical and microbiological profile of pediatric microbial keratitis in Eastern India
Source: PLoS One. 2026 Oct 14;21(10):e0359943. doi: 10.1371/journal.pone.0359943 (PMC13667804; doi:10.1371/journal.pone.0359943)
Supplement: Peer review history

# Peer review: Decision Letter 0

**DOI:** 10.1371/journal.pone.0359943.r001

**Authors:** Deepak Shukla

15 Jul 2026

Dear Dr. Kusumesh,

plosone@plos.org. When you're ready to submit your revision, log on to https://www.editorialmanager.com/pone/ and select the 'Submissions Needing Revision' folder to locate your manuscript file.

- A letter that responds to each point raised by the academic editor and reviewer(s). You should upload this letter as a separate file labeled 'Response to Reviewers'.
- A marked-up copy of your manuscript that highlights changes made to the original version. You should upload this as a separate file labeled 'Revised Manuscript with Track Changes'.
- An unmarked version of your revised paper without tracked changes. You should upload this as a separate file labeled 'Manuscript'.

If you would like to make changes to your financial disclosure, please include your updated statement in your cover letter. Guidelines for resubmitting your figure files are available below the reviewer comments at the end of this letter.

If applicable, we recommend that you deposit your laboratory protocols in protocols.io to enhance the reproducibility of your results. Protocols.io assigns your protocol its own identifier (DOI) so that it can be cited independently in the future. For instructions see: https://journals.plos.org/plosone/s/submission-guidelines#loc-laboratory-protocols. Additionally, PLOS ONE offers an option for publishing peer-reviewed Lab Protocol articles, which describe protocols hosted on protocols.io. Read more information on sharing protocols at https://plos.org/protocols?utm_medium=editorial-email&utm_source=authorletters&utm_campaign=protocols.

As the corresponding author, your ORCID iD is verified in the submission system and will appear in the published article. PLOS supports the use of ORCID, and we encourage all coauthors to register for an ORCID iD and use it as well. Please encourage your coauthors to verify their ORCID iD within the submission system before final acceptance, as unverified ORCID iDs will not appear in the published article. *Only*  the individual author can complete the verification step; PLOS staff *cannot*  verify ORCID iDs on behalf of authors.

We look forward to receiving your revised manuscript.

Kind regards,

Deepak Shukla

Academic Editor

PLOS One

Journal Requirements:

When submitting your revision, we need you to address these additional requirements.

1. Please ensure that your manuscript meets PLOS ONE's style requirements, including those for file naming. The PLOS ONE style templates can be found at

https://journals.plos.org/plosone/s/file?id=wjVg/PLOSOne_formatting_sample_main_body.pdf and

https://journals.plos.org/plosone/s/file?id=ba62/PLOSOne_formatting_sample_title_authors_affiliations.pdf.

2. Thank you for stating in your Funding Statement:

“This study was funded by the Multi-Disciplinary Research Unit (MRU), Indira Gandhi Institute of Medical Sciences, Patna, Bihar, under LRAC-approved project support (sanction letter dated 05 April 2024)”

Please provide an amended statement that declares *all* the funding or sources of support (whether external or internal to your organization) received during this study, as detailed online in our guide for authors at http://journals.plos.org/plosone/s/submit-now.  Please also include the statement “There was no additional external funding received for this study.” in your updated Funding Statement.

Please include your amended Funding Statement within your cover letter. We will change the online submission form on your behalf.

3. Thank you for stating the following financial disclosure:

“This study was funded by the Multi-Disciplinary Research Unit (MRU), Indira Gandhi Institute of Medical Sciences, Patna, Bihar, under LRAC-approved project support (sanction letter dated 05 April 2024)”

Please state what role the funders took in the study.  If the funders had no role, please state: "The funders had no role in study design, data collection and analysis, decision to publish, or preparation of the manuscript."

If this statement is not correct you must amend it as needed.

Please include this amended Role of Funder statement in your cover letter; we will change the online submission form on your behalf.

4. Please provide a complete Data Availability Statement in the submission form, ensuring you include all necessary access information or a reason for why you are unable to make your data freely accessible. If your research concerns only data provided within your submission, please write "All data are in the manuscript and/or supporting information files" as your Data Availability Statement.

5. Please be informed that funding information should not appear in the Acknowledgments section or other areas of your manuscript. We will only publish funding information present in the Funding Statement section of the online submission form. Please remove any funding-related text from the manuscript.

6. Please include captions for your Supporting Information files at the end of your manuscript, and update any in-text citations to match accordingly. Please see our Supporting Information guidelines for more information: http://journals.plos.org/plosone/s/supporting-information.

7. If the reviewer comments include a recommendation to cite specific previously published works, please review and evaluate these publications to determine whether they are relevant and should be cited. There is no requirement to cite these works unless the editor has indicated otherwise.

[Note: HTML markup is below. Please do not edit.]

Reviewers' comments:

Reviewer's Responses to Questions

**Comments to the Author**

1. Is the manuscript technically sound, and do the data support the conclusions?

Reviewer #1: Yes

Reviewer #2: Partly

**********

2. Has the statistical analysis been performed appropriately and rigorously?

Reviewer #1: Yes

Reviewer #2: I Don't Know

**********

3. Have the authors made all data underlying the findings in their manuscript fully available?

The PLOS Data policy

Reviewer #1: Yes

Reviewer #2: Yes

**********

4. Is the manuscript presented in an intelligible fashion and written in standard English?

Reviewer #1: Yes

Reviewer #2: Yes

**********

Reviewer #1: Overall, this is a clinically relevant prospective study with a reasonably large pediatric cohort and an interesting age-stratified analysis. However, some issues should be addressed before publication. My comments are below.

1. The authors divided patients into ≤5 years and >5 years age groups, but the rationale for selecting 5 years as the cutoff is not adequately explained. Was this based on biological, developmental, nutritional, or epidemiological considerations? A stronger justification should be provided in the Methods section.

2. The reported prevalence of vitamin A deficiency (42.9 % in children ≤5 years) is remarkably high and represents one of the most important findings of the study. More details are needed regarding: Timing of serum retinol measurement and Laboratory methods used for retinol assessment.

3. The study identifies several factors associated with severe disease and surgical intervention. However, only univariate comparisons are presented. A multivariate logistic regression evaluating predictors of Surgical intervention, Perforation and Poor outcome,

would substantially strengthen the conclusions.

4. Visual acuity outcomes were only reported for children >5 years. Since amblyopia risk is a major concern in younger children, the authors should discuss alternative methods used to assess visual function in preverbal children and acknowledge this limitation more explicitly.

5. The definition of poor outcome includes surgical intervention. However, surgery may represent successful management rather than treatment failure. The authors should justify this definition.

6. The follow-up period was limited to 3 months. Longer-term outcomes such as: Amblyopia, Graft survival, Corneal scarring, Visual rehabilitation, cannot be assessed. This limitation should be emphasized.

7. Table 2 is somewhat difficult to interpret because percentages appear to be calculated using different denominators. Please clarify whether percentages are based on total patients within each age group or total occurrences of risk factors.

8. Several sentences require language editing. Examples include:

• "It is prospective study with large number of children" → "It is a prospective study with a large number of children."

• "microbiological workup in is often challenging" → "microbiological workup is often challenging."

• "children ≤5 years exhibiting more than one systemic risk factor" → "children ≤5 years exhibited more than one systemic risk factor."

Language editing is recommended before publication.

Overall Recommendation

Major Revision

The study addresses an important topic and provides valuable data from an underrepresented region. However, some changes are needed before the manuscript can be considered for publication.

Reviewer #2: The manuscript entitled ‘Age-related differences in the clinical and microbiological profile of pediatric microbial keratitis in Eastern India’, aims to evaluate the demographics, predisposing factors, microbiological profile, management and outcomes of pediatric microbial keratitis in Eastern India with emphasis on age-related differences.

Please see the attached review report for detailed comments.

**********

what does this mean?). If published, this will include your full peer review and any attached files.

If you choose “no”, your identity will remain anonymous but your review may still be made public.

**Do you want your identity to be public for this peer review?**  For information about this choice, including consent withdrawal, please see our Privacy Policy

Reviewer #1: **Yes:**  DIVYA KAPOOR

Reviewer #2: No

**********

[NOTE: If reviewer comments were submitted as an attachment file, they will be attached to this email and accessible via the submission site. Please log into your account, locate the manuscript record, and check for the action link "View Attachments". If this link does not appear, there are no attachment files.]

To ensure your figures meet our technical requirements, please review our figure guidelines: https://journals.plos.org/plosone/s/figures

You may also use PLOS’s free figure tool, NAAS, to help you prepare publication quality figures: https://journals.plos.org/plosone/s/figures#loc-tools-for-figure-preparation.

NAAS will assess whether your figures meet our technical requirements by comparing each figure against our figure specifications.

Attachment Submitted filename: Final Review Report_PONE-D-26-21297.pdf

# Author comment: Author response to Decision Letter 1

**DOI:** 10.1371/journal.pone.0359943.r002

4 Sep 2026

Manuscript PONE-D-26-21297

Academic Editor: Dr. Deepak Shukla

Dear Dr. Shukla,

We sincerely thank you and the reviewers for the careful and constructive evaluation of our manuscript, “Age-related differences in the clinical and microbiological profile of pediatric microbial keratitis in Eastern India.” We are grateful for the opportunity to revise the manuscript and have carefully addressed all comments raised by the Academic Editor and reviewers.

Our point-by-point responses are provided below, with each comment reproduced in bold followed by our response. All corresponding revisions have been incorporated into the revised manuscript and are shown in revised manuscript with tracked changes. All changes are highlighted in red for ease of reference.

Comments to the Authors:

Academic editor

1. Please ensure that your manuscript meets PLOS ONE's style requirements, including those for file naming. The PLOS ONE style templates can be found at

https://journals.plos.org/plosone/s/file?id=wjVg/PLOSOne_formatting_sample_main_body.pdf and

https://journals.plos.org/plosone/s/file?id=ba62/PLOSOne_formatting_sample_title_authors_affiliations.pdf.

Response: We thank the Academic Editor for this guidance. The revised files have been prepared according to the journal’s prescribed templates.

2. Thank you for stating in your Funding Statement:

“This study was funded by the Multi-Disciplinary Research Unit (MRU), Indira Gandhi Institute of Medical Sciences, Patna, Bihar, under LRAC-approved project support (sanction letter dated 05 April 2024)”

Please provide an amended statement that declares *all* the funding or sources of support (whether external or internal to your organization) received during this study, as detailed online in our guide for authors at http://journals.plos.org/plosone/s/submit-now. Please also include the statement “There was no additional external funding received for this study.” in your updated Funding Statement.

Please include your amended Funding Statement within your cover letter. We will change the online submission form on your behalf.

Response: Thank you for the clarification. We have amended the Funding Statement to disclose the complete source of internal financial support received for this study, including the sanction details and sanctioned amount. We have also confirmed that no additional external funding was received. The revised statement has been included in the cover letter as requested.

We have added the following Funding statement to the revised cover letter:

“This study was funded by the Multi-Disciplinary Research Unit (MRU), Indira Gandhi Institute of Medical Sciences, Patna, Bihar, under LRAC-approved project support. As per the sanction letter dated 05/04/2024, a sum of Rs. 2,652 USD was approved for the project. The funder had no role in study design, data collection and analysis, decision to publish, or preparation of the manuscript. There was no additional external funding received for this study.” (Cover letter)

3. Thank you for stating the following financial disclosure:

“This study was funded by the Multi-Disciplinary Research Unit (MRU), Indira Gandhi Institute of Medical Sciences, Patna, Bihar, under LRAC-approved project support (sanction letter dated 05 April 2024)”

Please state what role the funders took in the study. If the funders had no role, please state: "The funders had no role in study design, data collection and analysis, decision to publish, or preparation of the manuscript."

Response: Thank you for this clarification. We have added the following role of the funder statement to the revised cover letter:

“The funder had no role in study design, data collection and analysis, decision to publish, or preparation of the manuscript.” (Cover letter)

4. Please provide a complete Data Availability Statement in the submission form, ensuring you include all necessary access information or a reason for why you are unable to make your data freely accessible. If your research concerns only data provided within your submission, please write "All data are in the manuscript and/or supporting information files" as your Data Availability Statement.

Response: Thank you for this comment. We have updated the Data Availability Statement in the submission form as follows:

“All data are in the manuscript and/or Supporting Information files.”

5. Please be informed that funding information should not appear in the Acknowledgments section or other areas of your manuscript. We will only publish funding information present in the Funding Statement section of the online submission form. Please remove any funding-related text from the manuscript.

Response: Thank you. We have removed the funding information from the Acknowledgments and retained it only in the cover letter

6. Please include captions for your Supporting Information files at the end of your manuscript, and update any in-text citations to match accordingly. Please see our Supporting Information guidelines for more information: http://journals.plos.org/plosone/s/supporting-information.

Response: Thank you. We have added captions for all Supporting Information files at the end of the manuscript and updated the corresponding in-text citations accordingly. (legends of supporting file, Page 27, lines 544-545)

“Legends of supporting files:

S1 Protocol. Approved study proposal for pediatric microbial keratitis research.

S2 Dataset. De-identified study dataset.”

7. If the reviewer comments include a recommendation to cite specific previously published works, please review and evaluate these publications to determine whether they are relevant and should be cited. There is no requirement to cite these works unless the editor has indicated otherwise.

Response: We thank the Academic Editor for this guidance. Relevant studies have been cited where appropriate, while no references were added solely in response to reviewer suggestions when they were not directly pertinent to the present study.

Reviewer #1:

Overall, this is a clinically relevant prospective study with a reasonably large pediatric cohort and an interesting age-stratified analysis. However, some issues should be addressed before publication.

Response: We thank the reviewer for the positive comments. We have addressed all the points raised and revised the manuscript accordingly.

1. The authors divided patients into ≤5 years and >5 years age groups, but the rationale for selecting 5 years as the cutoff is not adequately explained. Was this based on biological, developmental, nutritional, or epidemiological considerations? A stronger justification should be provided in the Methods section.

Response: We thank the reviewer for this important observation and agree that the rationale for selecting 5 years as the cutoff required clarification. Pediatric microbial keratitis is not a homogeneous entity across childhood. Children aged ≤5 years are largely preverbal or preschool-aged, have limited cooperation during examination, and are more vulnerable to nutritional deficiencies and systemic illnesses. In contrast, children aged >5 years are generally school-going, more cooperative, and allow more reliable history taking and visual assessment. Thus, 5 years was considered a clinically and developmentally meaningful cutoff for subgroup comparison. This rationale has now been added to the introduction and results section.

“Patients were divided into two age groups, ≤5 years and >5 years, based on recognized developmental differences in communication ability, cooperation during examination, and nutritional vulnerability, which may influence disease presentation, management, and outcomes.” (Result; demographics, page 7. 8, lines 157-160)

2. The reported prevalence of vitamin A deficiency (42.9 % in children ≤5 years) is remarkably high and represents one of the most important findings of the study. More details are needed regarding: Timing of serum retinol measurement and Laboratory methods used for retinol assessment.

Response: We thank the reviewer for highlighting this important finding and for suggesting that further details of serum retinol assessment be provided. In children with suspected vitamin A deficiency, serum retinol was measured at admission, before vitamin A supplementation. Laboratory estimation was performed using high-performance liquid chromatography (HPLC). Vitamin A deficiency was defined as a serum retinol level <0.20 mg/L, corresponding to the WHO criterion of <0.70 µmol/L. These details have now been added to the Methods section.

“In children with suspected vitamin A deficiency, Serum retinol levels were measured at admission, prior to treatment initiation, using High-Performance Liquid Chromatography (HPLC). Vitamin A deficiency was defined as serum retinol <0.20 mg/L (<0.70 µmol/L) per WHO criteria.” (Materials and methods, page 6, lines 121-124)

Serum retinol values are reported in mg/L throughout the manuscript.

“The median serum retinol level was 0.03 mg/L (IQR 0.02–0.13), which increased to 0.22 mg/L (IQR 0.20–0.27) following supplementation.” (Results; Predisposing factors, page 10, paragraph 1, lines 189-190)

3. The study identifies several factors associated with severe disease and surgical intervention. However, only univariate comparisons are presented. A multivariate logistic regression evaluating predictors of Surgical intervention, Perforation and Poor outcome,

would substantially strengthen the conclusions.

Response: We thank the reviewer for this valuable suggestion. Multivariable logistic regression analyses were performed to identify independent predictors of surgical intervention, perforation at presentation, and poor outcome.

• We first revised the definition of poor outcome in the Materials and Methods section as follows:

“At the end of 3 months, successful outcome was defined as complete resolution of keratitis with corneal opacity, with or without prior surgical intervention, indicating successful control of infection, preservation of globe integrity, and potential for future visual rehabilitation. Poor outcome was defined as anterior staphyloma, phthisis bulbi, or primary graft failure during the study period. Anterior staphyloma and phthisis bulbi represent permanent structural failure with irreversible loss of ocular function. Primary graft failure was included because it indicated failure of visual rehabilitation during the study period, although regrafting may offer future visual potential.” (Materials and methods; outcome measures, page 6,7, lines 134-141)

• The Statistical Analysis section was revised as follows:

“To identify independent predictors of surgical intervention, perforation at presentation, and poor outcome, multivariable binary logistic regression was performed. The number of variables was limited according to the number of outcome events. Results are reported as odds ratio (OR)/adjusted odds ratio (aOR) with 95% confidence intervals. P<0.05 was considered statistically significant. “ (Statistical analysis, page 7, lines 147-152)

• The statistical significance threshold of P<0.05 has also been applied consistently throughout the manuscript.

• The Results section was also revised and regression results have also been added as a new table in the revised manuscript.

“On multivariable analysis, larger ulcer size and longer disease duration were independently associated with surgical intervention. Each 1-mm increase in ulcer size increased the odds of surgical intervention by 73% (aOR 1.73; 95% CI 1.05–2.85; p=0.032), while each additional day of disease increased the odds by 12% (aOR 1.12; 95% CI 1.03–1.22; p=0.009). Severe ulcer grade and longer disease duration were independently associated with perforation at presentation. Severe ulcers had more than sixfold higher odds of perforation (aOR 6.32; 95% CI 2.14–18.63; p=0.001), and each additional day of disease increased the odds by 14% (aOR 1.14; 95% CI 1.05–1.24; p=0.002). Severe ulcer grade (aOR 7.43; 95% CI 2.08–26.49; p=0.002) and incomplete immunization (aOR 4.25; 95% CI 1.28–14.07; p=0.018) were independently associated with poor outcome at 3 months.” (Results, page 14, lines 244-253)

4. Visual acuity outcomes were only reported for children >5 years. Since amblyopia risk is a major concern in younger children, the authors should discuss alternative methods used to assess visual function in preverbal children and acknowledge this limitation more explicitly.

Response: We thank the reviewer for this important comment. As stated in methods section, in preverbal or poorly cooperative children (≤5 years), visual function was assessed using fixation and following of light, whereas Snellen or picture-chart visual acuity was recorded in cooperative children (>5 years). However, fixation and following responses are qualitative assessments that cannot be reliably converted to logMAR values or directly compared with quantitative visual acuity measurements in older children. Additionally, we acknowledge that our 3-month follow-up period was insufficient to capture long-term visual recovery and the development of deprivation amblyopia, which is a major concern in children ≤5 years. This has been explicitly added as a limitation in the Discussion section.

“In addition, quantitative visual acuity assessment was not possible in many younger children, in whom visual function was assessed qualitatively by fixation and following of light. Therefore, visual outcomes could not be directly compared between age groups. Deprivation amblyopia, particularly in children aged ≤5 years, could not be adequately assessed during the short follow-up period.” (Discussion, page 21, lines 393-399)

5. The definition of poor outcome includes surgical intervention. However, surgery may represent successful management rather than treatment failure. The authors should justify this definition.

Response: We thank the reviewer for this important observation. We agree that surgical intervention should not itself be considered a poor outcome, as surgery may successfully control infection and preserve globe integrity. We have therefore revised the definition of outcome in the Methods section.

“At the end of 3 months, successful outcome was defined as complete resolution of keratitis with corneal opacity, with or without prior surgical intervention, indicating successful control of infection, preservation of globe integrity, and potential for future visual rehabilitation. Poor outcome was defined as anterior staphyloma, phthisis bulbi, or primary graft failure during the study period. Anterior staphyloma and phthisis bulbi represent permanent structural failure with irreversible loss of ocular function. Primary graft failure was included because it indicated failure of visual rehabilitation during the study period, although regrafting may offer future visual potential.” (Materials and methods; outcome measures, page 7, lines 134-141)

6. The follow-up period was limited to 3 months. Longer-term outcomes such as: Amblyopia, Graft survival, Corneal scarring, Visual rehabilitation, cannot be assessed. This limitation should be emphasized.

Response: We thank the reviewer for this important comment. We agree that the 3-month follow-up period was insufficient to assess long-term graft survival, corneal scarring, visual rehabilitation, and deprivation amblyopia. This limitation has now been explicitly added to the Discussion section.

“Deprivation amblyopia, particularly in children aged ≤5 years, could not be adequately assessed during the short follow-up period. The 3-month follow-up period also limited assessment of longer-term outcomes including corneal graft survival, progression of corneal scarring, and visual rehabilitation.” (Discussion, page 21, paragraph 1, lines 396-399)

7. Table 2 is somewhat difficult to interpret because percentages appear to be calculated using different denominators. Please clarify whether percentages are based on total patients within each age group or total occurrences of risk factors.

Response: We thank the reviewer for this observation. We have clarified that all percentages in Table 2 were calculated using the total number of children in each age group as the denominator: 42 children aged ≤5 years and 44 children aged >5 years. As some children had multiple predisposing factors, the percentages of individual factors may not total 100%. This clarification has now been added as a table footnote.

“Percentages were calculated using the total number of children in each age group as the denominator. As some children had more than one predisposing factor, the percentages for individual risk factors may not total 100%.” (Results, page 11, table footnote, lines 195-197)

Additionally, a typographical error in the fever percentage in the ≤5 years group has been corrected from 16 (29.0%) to 16 (38.1%). (Results, page 11, table 2)

8. Several sentences require language editing. Examples include:

• "It is prospective study with large number of children" → "It is a prospective study with a large number of children."

• "microbiological workup in is often challenging" → "microbiological workup is often challenging."

• "children ≤5 years exhibiting more than one systemic risk factor" → "children ≤5 years exhibited more than one systemic risk factor."

Response: We thank the reviewer for pointing this out. The manuscript has been carefully revised for grammar and language, including the sentences highlighted by the reviewer, and similar errors have been corrected throughout the manuscript.

“It is a prospective study with large number of children” (Discussion, page 21, lines 403)

“microbiological workup is often challenging in children” (Discussion, page 19, lines 364)

“children ≤5 years exhibited more than one systemic risk factor.” (Results, Predisposing factors, page 10, , lines 187)

Reviewer 2

Comments:

The manuscript entitled ‘Age-related differences in the clinical and microbiological profile of pediatric microbial keratitis in Eastern India’, aims to evaluate the demographics, predisposing factors, microbiological profile, management and outcomes of pediatric microbial keratitis in Eastern India with emphasis on age-related differences.

Following comments need to be addressed at appropriate places in the manuscript:

Response: We thank the reviewer for reviewing our manuscript and for the constructive comments. We have addressed each point raised and revised the manuscript accordingly.

1. How is this study different from the author’s own previous study (Kusumesh et al., IJO, 2023, PMID: 37870015)? Please highlight in terms of novelty, new information, and distinctions.

Response: We thank the reviewer for this important comment. Our 2023 study was a retrospective and all-age (2,303 patients, mean age 48.4 years), in which in which children constituted only a small proportion of the cohort (<10 years, 0.4%; 11–20 years, 2.9%) and were not analyzed separately. In contrast, the present prospective study exclusively evaluates children ≤14 years and specifically compares those aged ≤5 years with older children. It additionally assesses pediatric-specific factors including nutritional status, vitamin A deficiency, immunization, bilateral involvement, 3-month outcomes, and independent predictors of surgery, perforation, and poor outcome. These distinctions and the novelty of the present study have now been highlighted in the Discussion.

“Unlike our previous retrospective study, which evaluated microbial keratitis across all age groups and focused mainly on regional epidemiology and microbiology, the present study exclusively included children and systematically assessed pediatric-specific factors such as nutritional status, vitamin A deficiency, immunization, bilateral involvement, management, and outcomes.” (Discussion, page 15, lines 265-269)

2. Why were the children categorized in >5 and <5 years? The rationale of choosing 5 years of age as a bar is not clear.

Response: We thank the reviewer for this important comment. As also clarified in response to Reviewer 1, the cutoff of 5 years was chosen because it separates two clinically and developmentally distinct pediatric groups. Children aged ≤5 years are largely preverbal or preschool-aged, have limited cooperation during examination, and are more vulnerable to nutritional deficiencies and systemic illnesses. In contrast, children aged >5 years are generally school-going, more cooperative, and allow more reliable history taking and visual assessment. Therefore, 5 years was considered a clinically and developmentally meaningful cutoff for subgroup comparison. This rationale has now been added to the introduction and Methods section.

“Patients were divided into two age groups, ≤5 years and >5 years, based on recognized developmental differences in communication ability, cooperation during examination, and nutritional vulnerability, which may influence disease presentation, management, and outcomes.” (Result; demographics, page 7. 8, lines 157-160)

3. Line 41/Materials and methods: ‘This prospective study…….suspected microbial keratitis…….’. Why were the suspected and not only the confirmed cases considered? Was this suspicion based on clinical or microbiological profiling of patients? Ideally, only the microbiologically and clinically confirmed cases should only be included in this study.

Response: We thank the reviewer for this important comment. Microbial keratitis was diagnosed clinically based on the presence of corneal stromal infiltrate with an overlying epithelial defect with or without hypopyon. Children fulfilling these clinical criteria were included in the study and corneal scrapings were subsequently sent for smear and culture. Microbiological confirmation was not used as an inclusion criterion because many true cases of microbial keratitis may have negative smear or culture results, especially after prior antibiotic treatment. Restricting inclusion to microbiologically positive cases would therefore have excluded clinically definite cases and would not have represented the full spectrum of pediatric microbial keratitis. Based on the reviewer’s suggestion and for clarity, we have replaced “suspected microbial keratitis” with “clinically diagnosed microbial keratitis” throughout the manuscript, abstract and clarified the diagnostic criteria in the Methods section.

“This prospective study included children with clinically diagnosed microbial keratitis between April 2024 and September 2025.” (Abstract, page 2, lines 32)

“Children with clinically diagnosed microbial keratitis, defined by the presence of a corneal stromal infiltrate with an overlying epithelial defect with or without hypopyon, were recruited from the cornea clinic and emergency services.” (Materials and methods, page 4, lines 81-83)

4. Line 48/ Results: How do undernutrition and vitamin A deficiency aid to microbial infections?

Response: We thank the reviewer for this important comment. Vitamin A deficiency compromises corneal and conjunctival epithelial integrity and mucus-secreting function, thereby weakening the ocular-surface barrier and local defence against infection. Severe deficiency may lead to xerosis, ulceration, and keratomalacia, with subsequent secondary microbial infection. Undernutrition further impairs systemic immunity and tissue healing, increasing susceptibility to infection. These mechanisms and relevant supporting literature have now been added to the Discussion.

“VAD emerged as an important finding in this study. Despite supplementation programs, it remains an important cause of childhood blindness in children <5 years, with reported prevalence up to 30% in vulnerable population.27-30 In our study, 43% of children <5 years had VAD, with a median serum retinol level of 0.03 mg/L (IQR 0.02–0.13) at presentation. The high burden of VAD in these infants may be partly related to nutritional vulnerability caused by inappropriate feeding practices including bottle-feeding with diluted cow’s milk. The association of VAD with diarrhea and febrile illness is important as these illnesses can further deplete vitamin A stores and trigger acute ocular disease.31 This predominance of VAD in younger children is also reported by the recent study by Baker et al., in which the median age was 2.78 years, 95% children had bilateral involvement, and more than half showed keratomalacia with or without bacterial keratitis.32

Vitamin A is essential for maintaining corneal and conjunctival epithelial integrity and normal mucus-secreting function. Its deficiency causes ocular-surface dryness and xerosis with loss of local corneal defense. Severe deficiency may lead to ulceration and keratomalacia, which can become secondarily infected and result in stromal melting, perforation, and scarring. 33-36 Baker et al., in a retrospective study of children with VAD-related corneal disease requiring keratoplasty, demonstrated the severe consequences of this process, including keratomalacia, corneal perforation, anterior staphyloma, phthisis, and extensive corneal scarring.32

Undernutrition was also more frequent in younger children and is closely linked to vitamin A deficiency. Together, undernutrition and vitamin A deficiency may impair systemic immunity, epithelial integrity, and tissue healing, thereby increasing susceptibility to microbial keratitis and its severe complications. In addition, other micronutrients such as zinc, iron, folate, vitamin B12, and vitamin C also contribute to normal immune function and host resistance to infection.37 Incomplete immunization, observed in 32.6% of children in our study, may further reflect this systemic vulnerability. Among vaccine-preventable illnesses, measles is particularly important, as it can worsen vitamin A deficiency, suppress local and systemic immunity, and predispose to corneal ulceration and secondary microbial infection.31, 38 (Discussion, page 16, 17, lines 291-317)

31. Foster A, Sommer A. Corneal ulceration, measles, and childhood blindness in Tanzania. Br J Ophthalmol. 1987 May;71(5):331-43. doi: 10.1136/bjo.71.5.331. PMID: 3580349; PMCID: PMC1041162.

32. Baker DR, Misra DK, Achanta DSR, Edward DP, Ramappa M. Clinical and histologic characterization of vitamin A deficiency in children undergoing penetrating keratoplasty. Int Ophthalmol. 2025 Aug 12;45(1):331. doi: 10.1007/s10792-025-03703-2. PMID: 40794322.

33. Sommer A. Xerophthalmia and vitamin A status. Prog Retin Eye Res. 1998 Jan;17(1):9-31. doi: 10.1016/s1350-9462(97)00001-3. PMID: 9537797.

34. Semba RD. Vitamin A, immunity, and infection. Clin Infect Dis. 1994 Sep;19(3):489-99. doi: 10.1093/clinids/19.3.489. PMID: 7811869.

35. Sommer A. Vitamin A Deficiency and Its Consequences: A Field Guide to Detection and Control. 3rd ed. Geneva: World Health Organization; 1995.

36. Sharma S. Keratitis. Biosci Rep. 2001 Aug;21(4):419-44. doi: 10.1023/a:1017939725776. PMID: 11900320

(References, page 25, 26, lines 502-513)

5. Line 60: What could be the possible reasons for children with age more than 5 years to get fungal infections than bacterial keratitis?

Response: We thank the reviewer. This age-related shift likely reflects differences in predisposing factors. Trauma with vegetative or organic matter is an important risk factor for fungal keratitis in tropical regions. Children >5 years are more ambulatory, school-going, and exposed to outdoor activities, increasing the risk of corneal trauma, whereas younger children are more often supervised indoors. This is consistent with our findings, in which ulcers in older children were more frequently trauma-related and fungal in etiology. This clarification has now been clarified in the Discussion.

“This age-related difference in trauma exposure may also explain the predominance of fungal over bacterial keratitis in older children. Injury with vegetative or organic matter, an important risk factor for fungal keratitis in tropical regions, is more likely in school-going children with greater outdoor exposure. .” (Discussion, page 17,18, lines 320-323)

6. Lines 122-125: Immunization and nutritional assessment: Considering that immunization and nutritional assessment are the important criteria, can the authors name a few vaccines and few nutritional elements which can be critical for occurrence, severity, and/or recurrence of microbial keratitis?

Response: We thank the reviewer for this important comment. In our study, 32.6% of children had incomplete immunization. Among vaccines, measles vaccination is particularly relevant as measles can reduce vitamin A levels, damage the corneal epithelium, and impair local and systemic immunity, thereby increasing susceptibility to corneal ulceration and secondary microbial infection. Among nutritional factors, vitamin A and overall nutritional status are especially important while micronutrients such as zinc, iron, folate, vitamin B12, and vitamin C also influence host immunity and susceptibility to infection. These aspects have now been clarified in the Discussion.

“Undernutrition was also more frequent in younger children and may further increase susceptibility to infection by impairing systemic immunity and tissue healing. In addition to vitamin A, other micronutrients such as zinc, iron, folate, vitamin B12, and vitamin C also contribute to normal immune function and host resistance to infection. (1) Incomplete immunization, observed in 32.6% of children in our study, may further reflect this systemic vulnerability. Among vaccine-preventable illnesses, measles is particularly important because it can worsen vitamin A deficiency, suppress local and systemic immunity, damage the corneal epithelium, and predispose to corneal ulceration and secondary microbial infection.” (Discussion, page 17, lines 309-317)

37. Bhaskaram P. Micronutrient malnutrition, infection, and immunity: an overview. Nutr Rev. 2002 May;60(5 Pt 2):S40-5. doi: 10.1301/00296640260130722. PMID: 12035857.

31. Foster A, Sommer A. Corneal ulceration, measles, and childhood blindness in Tanzania. Br J Ophthalmol. 1987 May;71(5):331-43. doi: 10.1136/bjo.71.5.331. PMID: 3580349; PMCID: PMC1041162

38. Gupta BM, Singh M. Mortality and morbidity pattern in measles in Tanga District, Tanzania. Trop Geogr Med. 1975 Dec;27(4):383-6. PMID: 1216318.

39. (References, page 25, 26, lines 514-517)

7. Lines 152-154: Results/Demographics: ‘A total of 99 eyes from 86 …….patients with suspected microbial keratitis’. How many of these were the confirmed (microbiologically) cases?

Response: We thank the reviewer and have clarified this in the Results. All 99 eyes underwent microbiological evaluation by smear and culture. A causative organism was demonstrated in 51 eyes (51.5%), which constitute the microbiologically confirmed cases; 48 eyes (48.5%) were negative on both smear and culture (unidentified/sterile). These details have now been clearly added to the Results.

“All 99 eyes underwent microbiological evaluation by smear and culture. Causative organisms were identified in 51.5%, constituting the microbiologically confirmed cases, while 48.5% were negative on both smear and culture (unidentified/sterile). All microbiologically confirmed eyes were smear-positive, of which 36.4% of all eyes were also culture-positive. Microbiological confirmation was higher in children older than 5 years (68.2%) than in those ≤5 years (38.2%; P = 0.004). “ (Results; clinical picture and microbial profile, page 12, lines 214-219)

8. Table 1: What was the interrelationship between gender, laterality, socio-economic status, nutritional status, and immunization status? For example: How many of the confirmed male patients (children) were unilateral, belonging to lower middle socio-economic status, were undernourished and incompletely immunized? Such correlations among different groups may further add to data interpretation and conclusion.

Response: We thank the reviewer for this valuable suggestion. As per suggestion, we further analyzed the relationships among gender, laterality, socioeconomic status, nutritional status, and immunization status. Gender was not significantly associated with laterality, nutritional status, or immunization status. Overall, 12 out of 86 children (14.0%) were both undernourished and incompletely immunized; among them, 7 (58.3%) had unilateral and 5 (41.7%) had bilateral keratitis. Of these 12 children, 6 (50.0%) belonged to the lower-middle, 4 (33.3%) to the upper-lower, 1 (8.3%) to the lower, and 1 (8.3%) to the upper-middle socioeconomic group. Bilateral involvement was significantly more frequent in undernourished children than in normally nourished children (37.5% vs 6.5%; P=0.001) and in incompletely immunized than completely immunized children (28.6% vs 8.6%; P=0.024). Incomplete immunization was also more frequent among undernourished children (50.0% vs 25.8%; P=0.042). A higher proportion of bilateral disease was observed in the lower socioeconomic categories; however, this association did not reach statistical significance on exact testing (P=0.053).relevant paragraph is added in results.

“Further analysis showed that bilateral involvement was significantly more frequent among undernourished than normally nourished children (37.5% vs 6.5%; P=0.001) and among incompletely than completely immunized children (28.6% vs 8.6%; P=0.024). Incomplete immunization was also more common among undernourished children (50.0% vs 25.8%; P=0.042). Overall, 12 children (14.0%) were both undernourished and incompletely immunized, of whom 5 (41.7%) had bilateral keratitis. Bilateral involvement was more frequent in lower socioeconomic groups, although this association did not reach statistical significance (P=0.053).” (Results; Predisposing factors, page 11, lines 198-205)

9. Table 3: ‘Causative microbe’ should preferably come before ‘Previous medical history’. Further, were the duration of prescribed antimicrobial drugs and corticosteroid also considered? If yes, what were the dose start and endpoints with respect to date of presentation, onset, and resolution of infection. How many of the patients followed the prescribed treatment regimen?

Response: We thank the reviewer for these helpful suggestions. Table 3 has been reordered so that “Causative microbe” now precedes “Previous medical history.”

Prior topical antimicrobial and corticosteroid use and the duration of symptoms before presentation were recorded. However, as most children had received variable treatment before referral, the exact drugs, doses, treatment duration, and start/stop dates could not be reliably verified and were therefore not analyzed. Pre-referral adherence could also not be accurately assessed. This information has now been mentioned in discussion.

“In this study, as most children were referred after receiving variable treatment elsewhere, details regarding the specific drugs, doses, duration of therapy, and treatment adherence could not be reliably ascertained from the referral records.” (Discussion, page 18, paragraph 3, lines 336-338)

10. Table 4: Names of bacterial and fungal isolates should follow the standard nomenclature pattern (in italics), as applicable, throughout the manuscript

Response: We thank the reviewer for pointing this out. The names of all bacterial and fungal isolates in Table 4 and throughout the manuscript have been revised according to standard scientific nomenclature, with genus and species names italicized as appropriate. (Table 4, page 13)

Additional journal requirement – De-identification of supporting information dataset

We thank the journal office for highlighting the need for further de-identification of the Supporting information dataset. The S2 dataset has been carefully reviewed and revised accordingly. “Original study identification codes were removed and replaced with arbitrary sequential participant IDs (P001–P086) and eye-level IDs (E001–E099). Exact age in months was replaced with broader age categories (<1, 1–5, 6–10, and 11–14), and detailed socioeconomic categories were collapsed into broader Middle and Lower groups to reduce the risk of indirect identification”.

The revised dataset contains no names, initials, hospital numbers, contact details, addresses, dates of birth, examination dates, location information, or other direct personal identifiers.. In addition, a separate data dictionary worksheet has been added within the S2 file (sheet 2) to explain variable coding and abbreviations and to indicate whether variables are recorded at the participant or eye level.

The revised fully de-identified dataset has been re-uploaded as S2. de-identified study dataset.

Yours sincerely,

Corresponding author

Rakhi Kusumesh

drkrakhi@yahoo.com

Attachment Submitted filename: Response to reviewers.docx

# Peer review: Decision Letter 1

**DOI:** 10.1371/journal.pone.0359943.r003

**Authors:** Deepak Shukla

22 Sep 2026

Age-related differences in the clinical and microbiological profile of pediatric microbial keratitis in Eastern India

PONE-D-26-21297R1

Dear Dr. Kusumesh,

We’re pleased to inform you that your manuscript has been judged scientifically suitable for publication and will be formally accepted for publication once it meets all outstanding technical requirements.

Within one week, you’ll receive an e-mail detailing the required amendments. When these have been addressed, you’ll receive a formal acceptance letter and your manuscript will be scheduled for publication.

An invoice will be generated when your article is formally accepted. Please note, if your institution has a publishing partnership with PLOS and your article meets the relevant criteria, all or part of your publication costs will be covered. Please make sure your user information is up-to-date by logging into Editorial Manager at Editorial Manager® and clicking the ‘Update My Information' link at the top of the page. For questions related to billing, please contact billing support.

If your institution or institutions have a press office, please notify them about your upcoming paper to help maximize its impact. If they’ll be preparing press materials, please inform our press team as soon as possible -- no later than 48 hours after receiving the formal acceptance. Your manuscript will remain under strict press embargo until 2 pm Eastern Time on the date of publication. For more information, please contact onepress@plos.org.

Kind regards,

Deepak Shukla

Academic Editor

PLOS One

Additional Editor Comments (optional):

Reviewers' comments:

Reviewer's Responses to Questions

**Comments to the Author**

Reviewer #1: All comments have been addressed

**********

2. Is the manuscript technically sound, and do the data support the conclusions?

Reviewer #1: Yes

**********

3. Has the statistical analysis been performed appropriately and rigorously?

Reviewer #1: Yes

**********

4. Have the authors made all data underlying the findings in their manuscript fully available?

The PLOS Data policy

Reviewer #1: Yes

**********

5. Is the manuscript presented in an intelligible fashion and written in standard English?

Reviewer #1: Yes

**********

Reviewer #1: (No Response)

**********

what does this mean?). If published, this will include your full peer review and any attached files.

If you choose “no”, your identity will remain anonymous but your review may still be made public.

**Do you want your identity to be public for this peer review?**  For information about this choice, including consent withdrawal, please see our Privacy Policy

Reviewer #1: No

**********

# Editor report: Acceptance letter

**DOI:** 10.1371/journal.pone.0359943.r004

**Authors:** Deepak Shukla

PONE-D-26-21297R1

PLOS One

Dear Dr. Kusumesh,

I'm pleased to inform you that your manuscript has been deemed suitable for publication in PLOS One. Congratulations! Your manuscript is now being handed over to our production team.

At this stage, our production department will prepare your paper for publication. This includes ensuring the following:

* All references, tables, and figures are properly cited

* All relevant supporting information is included in the manuscript submission,

* There are no issues that prevent the paper from being properly typeset

You will receive further instructions from the production team, including instructions on how to review your proof when it is ready. Please keep in mind that we are working through a large volume of accepted articles, so please give us a few days to review your paper and let you know the next and final steps.

Lastly, if your institution or institutions have a press office, please let them know about your upcoming paper now to help maximize its impact. If they'll be preparing press materials, please inform our press team within the next 48 hours. Your manuscript will remain under strict press embargo until 2 pm Eastern Time on the date of publication. For more information, please contact onepress@plos.org.

You will receive an invoice from PLOS for your publication fee after your manuscript has reached the completed accept phase. If you receive an email requesting payment before acceptance or for any other service, this may be a phishing scheme. Learn how to identify phishing emails and protect your accounts at https://explore.plos.org/phishing.

If we can help with anything else, please email us at customercare@plos.org.

Thank you for submitting your work to PLOS One and supporting open access.

Kind regards,

PLOS One Editorial Office Staff

on behalf of

Professor Deepak Shukla

Academic Editor

PLOS One
